# The effects of Jerusalem artichoke and fermented soybean powder mixture supplementation on blood glucose and oxidative stress in subjects with prediabetes or newly diagnosed type 2 diabetes

**DOI:** 10.1038/s41387-018-0052-y

**Published:** 2018-07-19

**Authors:** Hyeon Yeong Ahn, Minjoo Kim, Cho Rong Seo, Hye Jin Yoo, Sang-Hyun Lee, Jong Ho Lee

**Affiliations:** 1 Research Center for Silver Science, Institute of Symbiotic Life-TECH, Yonsei University, Seoul, Korea; 2 Department of Science for Aging, Graduate School of Yonsei University, Seoul, Korea; 3 Department of Food and Nutrition, Brain Korea 21 PLUS Project, College of Human Ecology, Yonsei University, Seoul, Korea; 4 Department of Family Practice, National Health Insurance Corporation Ilsan Hospital, Goyang, Korea; 5 Department of Food and Nutrition, National Leading Research Laboratory of Clinical Nutrigenetics/Nutrigenomics, College of Human Ecology, Yonsei University, Seoul, Korea; 6 Cardiovascular Research Institute, Yonsei University College of Medicine, Seoul, Korea

## Abstract

**Background/Objectives:**

The objective of this study was to evaluate the effect of supplementation with a Jerusalem artichoke and fermented soybean powder mixture on blood glucose and oxidative stress levels.

**Subjects/Methods:**

This randomized, double-blinded, placebo-controlled study was conducted on 60 subjects with impaired fasting glucose (IFG), impaired glucose tolerance (IGT), or newly diagnosed type 2 diabetes. The subjects were randomly assigned to either a group that ingested 40 g of a Jerusalem artichoke and fermented soybean powder mixture (19.45 g each) daily or a group that received a placebo for 12 weeks. Paired *t*-test and independent *t*-test were performed for comparisons within groups and between groups, respectively.

**Results:**

Supplementation with the Jerusalem artichoke and fermented soybean powder mixture reduced the levels of fasting glucose (*p* < 0.001) and FFAs (*p* = 0.034), glucose at 60 min (*p* = 0.004), glucose (*p* = 0.006) areas under the response curve (AUC), homeostasis model assessment-insulin resistance (*p* = 0.018), and the urinary 8-epi-prostaglandin F_2α_ (8-epi-PGF_2α_) level (*p* = 0.028). The changes (Δ) in urinary 8-epi-PGF_2α_, glucose at 60 min, 120 min, and AUC, FFAs at 0 min and AUC were significantly different between the two groups. In addition, Δ glucose at 120 min (*r* = 0.472, *p* = 0.027) and the Δ glucose AUC (*r* = 0.572, *p* = 0.005) were positively correlated with △ plasma malondialdehyde in the test group.

**Conclusions:**

The consumption of a Jerusalem artichoke and fermented soybean powder mixture for 12 weeks was effective for reducing postprandial glucose and oxidative stress level, particularly 8-epi-PGF_2α_, in subjects with IFG, IGT, or newly diagnosed type 2 diabetes.

## Introduction

Diabetes is a chronic metabolic disease that is common worldwide and is caused by reduced organ function, which results in insulin resistance (IR) or lack of insulin^1^. According to the World Health Organization, 347 million people worldwide had diabetes in 2013, and diabetes will be the 7th most common cause of death by 2030^2^. The prevalence of diabetes worldwide was 6.4% in 2010 and will increase to 7.7% by 2030^3^. According to the 2013 Korean National Health and Nutrition Examination Survey, the incidence of diabetes has increased 2% since 2012^4^. People with impaired fasting glucose (IFG), impaired glucose tolerance (IGT), or both are at high risk of progressing to type 2 diabetes, which is a risk factor for cardiovascular disease^5,6^. The progression of IFG or IGT to type 2 diabetes can be delayed or prevented by lifestyle changes and pharmacological interventions^7^. According to the American Diabetes Association, medical nutrition therapy is an effective method for the management of diabetes^8^.

Jerusalem artichoke is a root vegetable, and the main components include fructans (fructose molecules connected by *β*-2,1 bonds), specifically inulin and fructooligosaccharides^9^. Additionally, chicory is a main source of inulin-type fructans, which are commonly extracted from the *Compositae* family and are known as prebiotic dietary fibers. Inulin-type fructans resist hydrolysis by human small intestinal digestive enzymes due to the β-configuration of the anomeric C_2_ in its fructose monomer; thus, inulin-type fructans have been classified as non-digestible oligosaccharides^10,11^. The enhancement of gastrointestinal functions is a primary endpoint that benefits from inulin-type fructans and reduces gastrointestinal-related disease risks, such as intestinal infections, colon cancer, and obesity^12,13^. Moreover, inulin and fructooligosaccharides stimulate the immune system, reduce the synthesis of triglycerides and fatty acids in the liver, and decrease blood glucose levels^14^. Several studies revealed that inulin-type fructans supplementation modulate glycemic indices, lipid profile, antioxidant status^15^, and some inflammatory, immunologic markers^16^, and calcium homeostasis^17^ in type 2 diabetes patients. Fermented soybeans have antidiabetic effects in diabetic animals and humans^18–20^. A previous study reported that the combination of Jerusalem artichoke and fermented soy had complementary antidiabetic effects through the potentiation of insulinotropic action and the reduction of IR in diabetic rats^21^. Therefore, we hypothesized that these findings might be extrapolated to humans, and the aim of this study was to investigate the effect of Jerusalem artichoke and fermented soybean powder mixture supplementation on blood glucose and oxidative stress levels.

## Subjects and methods

### Study subjects

The study subjects were recruited from the outpatient clinics and the Health Service Center at the National Health Insurance Corporation Ilsan Hospital (Goyang, Korea) from June 2013 to December 2014. According to the glucose test results, 60 subjects with IFG (100 mg/dL ≤ fasting blood glucose ≤ 125 mg/dL), IGT [140 mg/dL ≤ 2 h blood glucose in the 75-g oral glucose tolerance test (OGTT) ≤ 199 mg/dL], or newly diagnosed type 2 diabetes were enrolled in this study. The aim of the study was carefully explained to all participants who provided written informed consent. Subjects were excluded if they had any diagnosis of cancer, renal disease, liver disease, chronic gastrointestinal disorder, or chronic alcoholism; were pregnant or breastfeeding; or had taken insulin agents or glucose-lowering medications for the past month. The protocol was approved by the Institutional Review Board of Yonsei University and National Health Insurance Corporation Ilsan Hospital and registered at http://www.clinicaltrials.gov (NCT02506582). Study subjects were randomly assigned to the test or placebo group. A computer-generated block randomization program assigned the groups (test group:placebo group = 1:1). The study sample size was determined using SAS 9.4 software (SAS Institute Inc., NC, USA). In an exploratory clinical trial, the postprandial glucose (PPG) level at 30 min decreased by 10.0 mg/dL in the test group compared with a reduction of 2.5 mg/dL in the placebo group. The sample size for this study was determined using the following parameters:

1. Superiority test
2. Level of significance, *α* = 0.05
3. *β* = 0.2, power=80%
4. Rates for participants in the test group and placebo group, *λ* = 1, *n*_*t*_ (participant number of the test group) = *λn*_*c*_ (participant number of the placebo group).
5. We hypothesized that the changes in PPG levels at 30 min for the test group (*μ*_*t*_) would be 10.0 mg/dL, and those changes for the placebo group (*μ*_*c*_) would be 2.5 mg/dL.
6. We assumed a standard deviation of 9.63 mg/dL for both the test group (*σ*_*t*_) and the placebo group (*σ*_*c*_).

$$H_0:\mu _t \le \mu _c\quad H_1:\mu _t > \mu _c$$

**Fig. 1:**
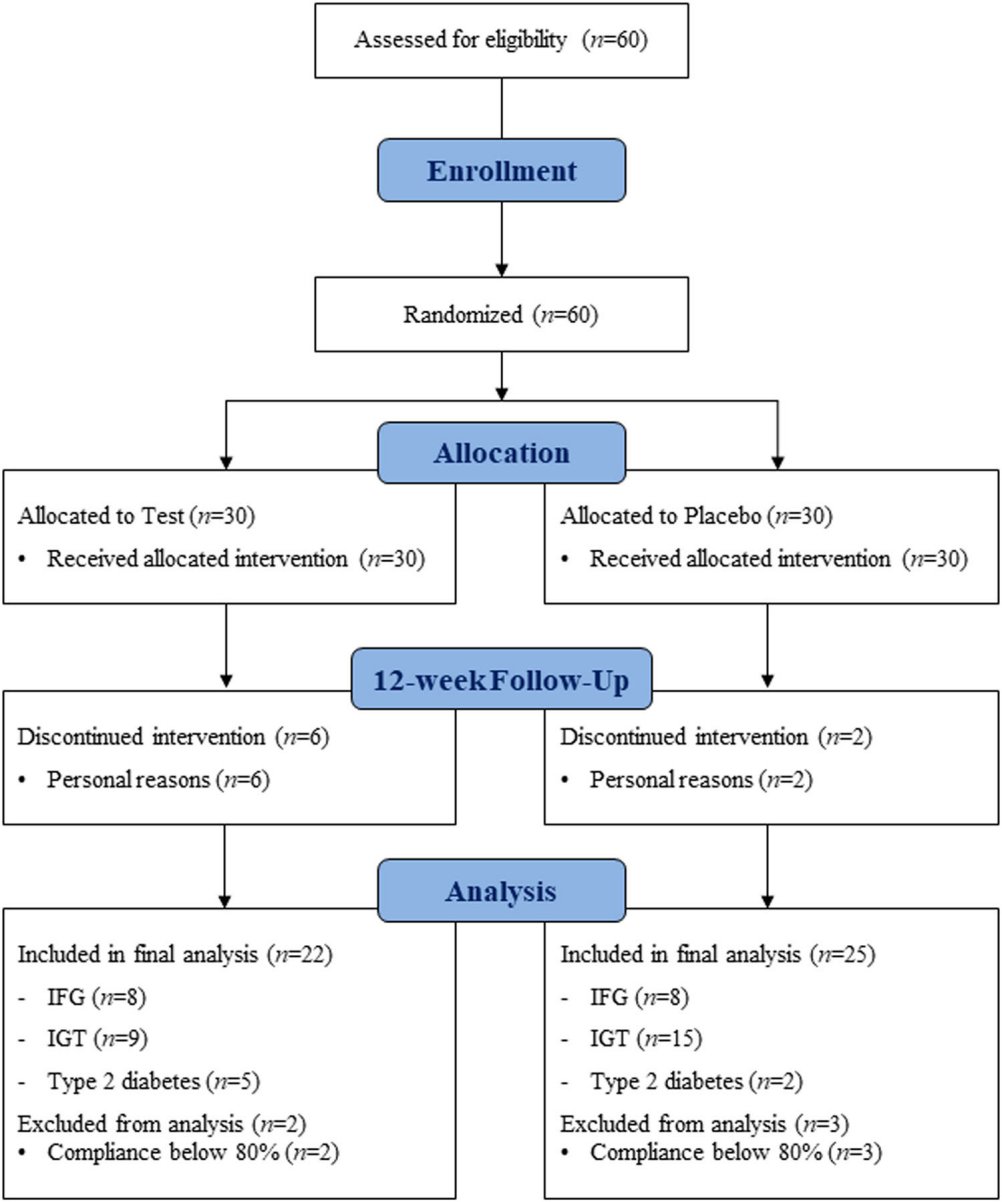
Flow diagram demonstrating the process for inclusion and analysis of randomized clinical trials

The sample size was 21 subjects per group. Assuming a dropout rate of 30%, we determined that 30 subjects should be recruited per group. Sixty subjects were enrolled, of whom eight dropped out after randomization due to personal reasons. Among 52 subjects who completed participation in the 12-week intervention study, five subjects who had less than 80% compliance were excluded from final analysis; the compliance was calculated as follows: Compliance (%) = Actual number of consumed supplements/Expected number of consumed supplements × 100. Finally, 47 subjects [placebo group (*n* = 25), test group (*n* = 22)] were included in the final analysis (Fig. 1).

### Study design and materials

After recruiting the study subjects, the entire study process was conducted at the Clinical Nutrigenetics/Nutrigenomics Lab in Yonsei University, Seoul, Korea. The study was a randomized, double-blinded, placebo-controlled trial. Subjects were divided into two groups. The test group consumed 40 g of a Jerusalem artichoke and fermented soybean powder mixture (3 times/day for 12 weeks) before each meal at home, and the placebo group consumed powdered rice flour (3 times/day for 12 weeks) before each meal at home; both the test and placebo products (powder) had low solubility, thus, we recommended the study subjects to take the powder first and then swallow it with water. The randomization list was blinded until the statistical analysis. The Jerusalem artichokes were sliced and steamed at 90–100 °C for 30 min, hot-air dried at 80 °C for 24 h and powdered. The soybeans were fermented with *Bacillus* spp. at 42 °C for 46 h, freeze-dried, and powdered. The 40 g Jerusalem artichoke and fermented soybean powder mixture (19.45 g each) contained 150 mg/g of inulin, which consisted of 29% fructooligosaccharides. The test product contained the following components: 97.27% Jerusalem artichoke and fermented soybean powder, 2.65% cornstarch, 0.07% caramel coloring, and 0.01% L-menthol. The placebo product contained the following components: 84.71% rice flour, 2.32% cornstarch, 0.003% caramel coloring, 0.01% L-menthol, and 12.957% water. The products were provided by Midari Farm (Yeongwol, Kangwon-Do, Korea).

### Anthropometric parameters, blood pressure, and blood and urine collection

All parameters were measured twice: at baseline (week 0) and follow-up (week 12). The body weight, height, and waist circumference were measured, and the body mass index (BMI) was determined as units of kilograms per square meter (kg/m^2^). Waist and hip circumferences were measured using a flexible measuring tape. Blood pressure (BP) was measured in the left arm with an automatic BP monitor (FT-200S, Jawon Medical, Gyeongsan, Korea). Blood and urine samples were also obtained at baseline and follow-up. After a 12-h fasting period, venous blood specimens were collected in ethylenediaminetetraacetate (EDTA)-treated tubes and plain tubes that were centrifuged to obtain plasma and serum. Urine was collected in polyethylene bottles containing 1% butylated hydroxytoluene. The collected samples were stored at −80 °C until analysis.

### OGTT and glucose-related biomarkers

To conduct the OGTT (at baseline and follow-up, respectively), we prepared a glucose–water solution. Briefly, 75 g of anhydrous glucose was dissolved in 300 mL of room temperature water. The solution was consumed by the study participants after a 12-h overnight fast, and a fasting venous blood specimen was collected (0 min). Venous blood samples were also collected at 30-min or 60-min intervals for 2 h (30, 60, and 120 min) in EDTA-treated tubes and plain tubes. Then, aliquots of the samples were stored at −80 °C prior to further analysis. Serum fasting glucose and blood glucose concentrations during the OGTT were measured using a hexokinase method with a Hitachi 7600 Autoanalyzer (Hitachi Ltd., Tokyo, Japan). Serum insulin was measured by an immunoradiometric assay kit from DIAsource ImmunoAssays S.A. (Louvain, Belgium). IR was calculated by the homeostasis model assessment (HOMA) method using the following equation: HOMA-IR = [Fasting insulin (μIU/mL) × Fasting glucose (mmol/L)]/22.5. Serum C-peptide was measured by an immunoradiometric assay with a C-peptide IRMA kit (Immunotech, Czech). Hemoglobin (HbA1c) was measured by an immunoturbidimetric analyzer with a turbidimeter.

### Serum lipid profile

Free fatty acids (FFAs) were measured using an enzymatic assay using the acyl-CoA synthetase-acyl-CoA oxidase method with a Hitachi 7600 Autoanalyzer (Hitachi Ltd., Tokyo, Japan). Triglycerides (TGs) and total cholesterol were measured with a Hitachi 7600 Autoanalyzer (Hitachi Ltd., Tokyo, Japan). High-density lipoprotein (HDL) cholesterol was measured using an enzymatic method. Low-density lipoprotein (LDL) cholesterol was calculated by the Friedewald formula: LDL cholesterol = total cholesterol−[HDL cholesterol  + (TG/5)].

### Urinary 8-epi-prostaglandin F_2α_ and plasma malondialdehyde

The urinary 8-epi-prostaglandin F_2α_ (8-epi-PGF_2a_) level was measured with a urinary isoprostane ELISA kit (Oxford Biomedical Research Inc., Rochester Hills, MI, USA). Urinary creatinine levels were determined using an alkaline picrate (Jaffe) reaction. Plasma malondialdehyde (MDA) was measured by determining the thiobarbituric acid-reactive substances (TBARS) with a TBARS Assay Kit (ZeptoMetrix Co., Buffalo, NY, USA).

### Dietary intake and total energy expenditure

The subjects were instructed to maintain their eating habits and a normal level of physical activity during the study period to ensure that any changes that were observed were not due to diet or physical activity. Nutrient intake was mainly calculated based on the 3-day dietary records (2 weekdays and 1 weekend day) using the Computer Aided Nutritional Analysis Program (CAN-pro 3.0, Korean Nutrition Society, Seoul, Korea). This record was completed at home after the study subjects received detailed explanations from well-trained dietitians. This measurement was repeated at weeks 0 and 12. In addition, the subjects completed a 24-h recall and a semi-quantitative food-frequency questionnaire with the assistance of a dietitian at weeks 0 and 12 to confirm the accuracy of the dietary record. Total energy expenditure (TEE) was calculated based on the basal metabolic rate (BMR), 24-h physical activity, and food-specific dynamic action, and the physical activity record was also completed at home on the same days that the dietary record was completed. The BMR was calculated using the Harris–Benedict equation. The 24-h physical activity was assessed by a physical activity questionnaire, developed based on the international physical activity questionnaire (IPAQ) and translated into Korean, at weeks 0 and 12; and all questions from the physical activity questionnaire were answered under the administration of interviewers to improve the accuracy of the survey.

### Primary and secondary outcomes

The primary outcome of this study was an improvement in the 30 min PPG level during the OGTT. The secondary outcomes were an improvement in the fasting glucose level at the 12-week follow-up compared to baseline levels; differences in the blood glucose levels (subtract baseline values from follow-up values) between groups at each time point during the OGTT; and other glucose-related biomarkers, including insulin, HOMA-IR, C-peptide, and HbA1c levels.

### Statistical analysis

Statistical analysis was performed with SPSS version 21.0 (IBM, Chicago, IL, USA). A paired *t*-test was conducted to compare the effect of supplementation within each group both before and after intervention. An independent *t*-test was conducted to compare the net change (Δ, difference from baseline) between the test and placebo groups. Pearson’s correlation coefficient was used to examine relationships between variables. A logarithmic transformation was performed on skewed variables. The results are expressed as the mean ± standard error (SE), and a *p*-value < 0.05 was considered statistically significant.

## Results

### Clinical and biochemical characteristics at baseline and changes of dietary intake

The clinical and biochemical characteristics of the placebo and test groups are presented in Table 1. No significant differences in baseline characteristics [i.e., age, sex distribution, height, weight, BMI, waist-to-hip ratio (WHR), BPs, serum lipid profiles, HbA1c, HOMA-IR, C-peptide, 8-epi-PGF_2α_, or MDA] were observed between the groups. The urinary 8-epi-PGF_2α_ level was significantly different between the two groups at baseline (*p* = 0.032). There were no significant differences observed in estimated total caloric intake (TCI), TEE, BMR, % carbohydrate intake, % protein intake, and % fat intake, and cholesterol intake at baseline and 12-week follow-up period between the test and placebo group (Table S1). In addition, the changed values of dietary intake and TEE did not show any significances between the two groups.

**Table 1:** Clinical and biochemical characteristics of the placebo and test groups at baseline

	Total (*n* = 47)				*p* ^a^	*p* ^b^	*p* ^c^	*p* ^d^
	Placebo group (*n* = 25)		Test group (*n* = 22)					
	Baseline	Follow-up	Baseline	Follow-up				
Age (years)	56.0 ± 1.28		54.4 ± 1.31		0.390			
Males/females, *n* (%)	10 (40.0)/15 (60.0)		4 (18.2)/18 (81.8)		0.102			
Height (cm)	161.6 ± 1.24		158.9 ± 1.38		0.154			
Weight (kg)	64.3 ± 1.72	64.2 ± 1.79	60.4 ± 2.35	60.2 ± 2.27	0.188	0.170		
Change	−0.03 ± 0.38		−0.19 ± 0.30				0.748	0.655
Body mass index (kg/m^2^)	24.6 ± 0.50	24.5 ± 0.50	23.8 ± 0.63	23.7 ± 0.60	0.336	0.300		
Change	−0.03 ± 0.14		−0.07 ± 0.12				0.838	0.672
Waist-to-hip ratio	0.90 ± 0.01	0.89 ± 0.01	0.88 ± 0.01	0.88 ± 0.01	0.387	0.466		
Change	0.00 ± 0.00		0.00 ± 0.00				0.505	0.613
Systolic BP (mmHg)	128.5 ± 2.69	119.6 ± 2.21^**^	127.4 ± 3.17	119.6 ± 3.26^*^	0.801	0.998		
Change	−8.88 ± 2.40		−7.84 ± 2.77				0.777	0.859
Diastolic BP (mmHg)	79.5 ± 1.72	75.8 ± 1.43^*^	82.2 ± 1.83	79.3 ± 2.47	0.291	0.214		
Change	−3.66 ± 1.36		−2.84 ± 1.83				0.717	0.469
Total cholesterol (mg/dL)^e^	193.7 ± 5.29	188.7 ± 6.28	201.6 ± 6.03	190.1 ± 6.17	0.320	0.846		
Change	−4.96 ± 4.56		−11.6 ± 5.45				0.355	0.535
HDL cholesterol (mg/dL)^e^	51.6 ± 1.91	51.6 ± 2.34	58.0 ± 2.77	56.1 ± 2.85	0.076	0.242		
Change	0.00 ± 1.79		−1.91 ± 1.37				0.411	0.677
LDL cholesterol (mg/dL)^e^	119.9 ± 4.90	114.9 ± 5.06	120.9 ± 6.38	112.4 ± 5.25	0.993	0.733		
Change	−4.95 ± 4.12		−8.45 ± 4.99				0.588	0.529
Triglycerides (mg/dL)^e^	110.9 ± 10.1	110.9 ± 11.5	114.0 ± 13.1	108.1 ± 10.6	0.976	0.967		
Change	−0.04 ± 12.5		−5.91 ± 13.2				0.748	0.732
Malondialdehyde (nmol/mL)^e^	8.68 ± 0.35	8.10 ± 0.33	8.33 ± 0.27	7.92 ± 0.42	0.685	0.634		
Change	−0.58 ± 0.35		−0.41 ± 0.43				0.758	0.976

Mean ± SE

^*^p < 0.05 and ^**^p < 0.01 derived from the paired t-test

^a^*p*-Values derived from the independent *t*-test for baseline values

^b^*p*-Values derived from the independent *t*-test for follow-up values

^c^*p*-Values derived from the independent *t*-test for change values

^d^*p*-Values adjusting for baseline

^e^Tested by logarithmic transformation

### The effects of 12 weeks of supplementation with a Jerusalem artichoke and fermented soybean powder mixture on blood glucose levels and blood glucose-related biomarkers

**Fig. 2:**
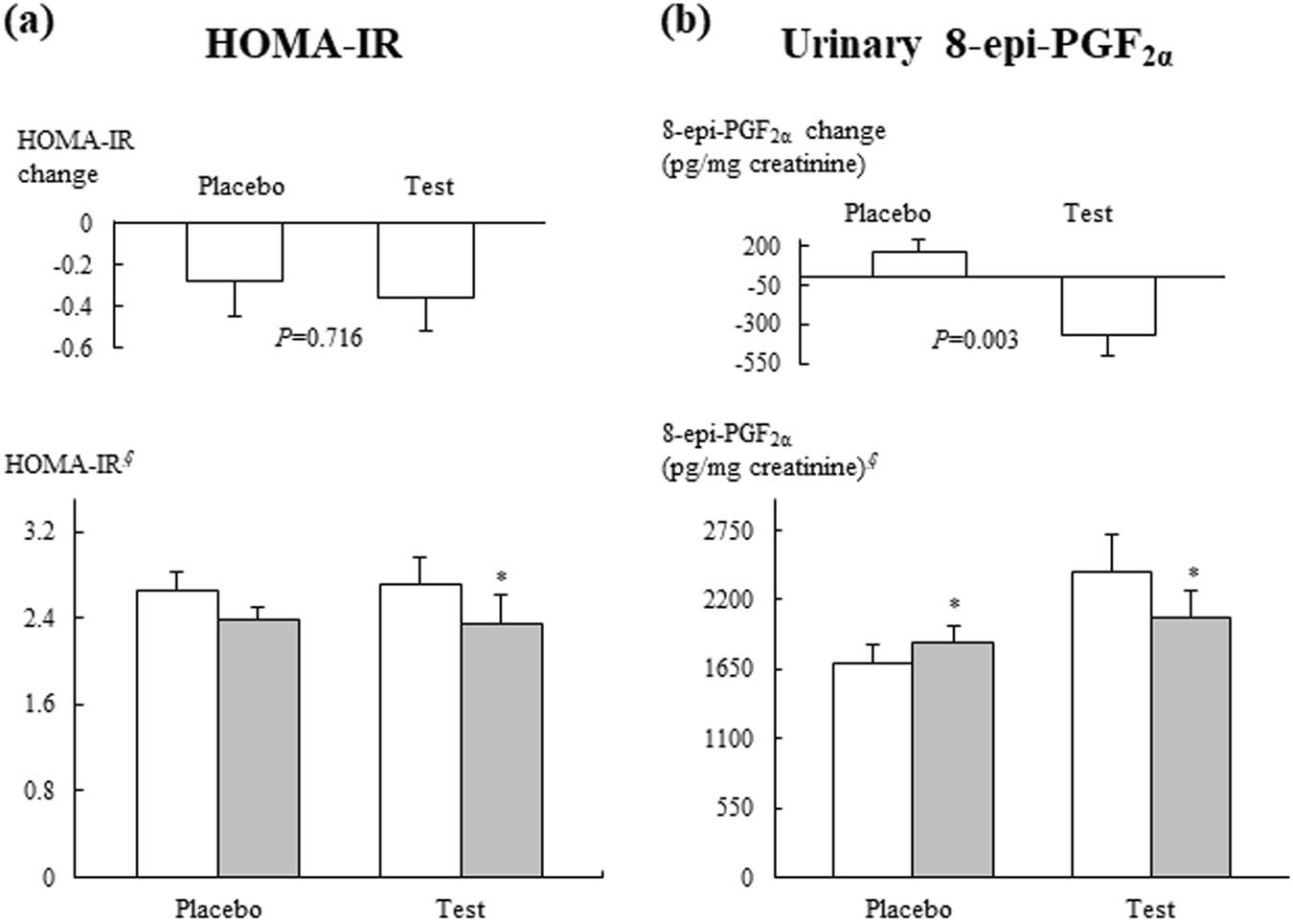
HOMA-IR and urinary 8-epi-PGF_2α_ levels at the initial visit (□) and the 12-week follow-up (■) and mean changes according to treatment. Mean ± SE. ^*§*^Tested by logarithmic transformation. *p*-Values were determined using the independent *t*-test. ^*^p < 0.05 compared with baseline values in each group as determined by the paired *t*-test

With the exception of BPs (Table 1), urinary 8-epi-PGF_2α_ levels (Fig. 2), blood glucose, and FFAs (Table 2), no significant mean changes in any of the tested clinical or biochemical characteristics were noted between the placebo and test groups. After 12 weeks of treatment, the test group exhibited significant reductions in blood glucose at 0 min (*p* = 0.001) and 60 min (*p* = 0.004) and glucose areas under the response curve (AUC) (*p* = 0.007) (Table 2). The Δ serum level of blood glucose at 60 min (*p* = 0.013) and 120 min (*p* = 0.021) and the glucose AUC (*p* = 0.012) in the test group significantly differed from the placebo group, furthermore, these significances were maintained after adjusting for baseline values.

**Table 2:** Blood glucose levels and glucose-related biomarkers of study participants

	Total (*n* = 47)				*p* ^a^	*p* ^b^	*p* ^c^	*p* ^d^
	Placebo group (*n* = 25)		Test group (*n* = 22)					
	Baseline	Follow-up	Baseline	Follow-up				
Glucose 0 min (mg/dL)^e^	107.6 ± 1.48	101.3 ± 1.73^**^	107.2 ± 1.68	97.7 ± 2.63^***^	0.839	0.193		
Change	−6.28 ± 1.78		−9.45 ± 1.90				0.230	0.210
Glucose 30 min (mg/dL)^e^	189.2 ± 6.36	185.6 ± 6.56	189.7 ± 9.73	186.8 ± 8.17	0.849	0.956		
Change	−3.56 ± 4.84		−2.91 ± 8.86				0.947	0.963
Glucose 60 min (mg/dL)^e^	192.3 ± 9.88	195.8 ± 9.51	205.2 ± 11.8	181.3 ± 12.0^**^	0.402	0.243		
Change	3.56 ± 7.28		−23.9 ± 7.74				**0.013**	**0.021**
Glucose 120 min (mg/dL)^e^	153.4 ± 8.71	166.5 ± 11.5	167.7 ± 11.2	154.0 ± 11.8	0.367	0.463		
Change	13.1 ± 8.22		−13.7 ± 7.51				**0.021**	**0.031**
Glucose AUC (mg/dL×h)^e^	342.4 ± 13.1	348.3 ± 14.5	359.4 ± 17.3	330.8 ± 16.3^**^	0.470	0.378		
Change	5.86 ± 9.05		−28.6 ± 9.45				**0.012**	**0.017**
FFAs 0 min (μEq/L)^e^	572.4 ± 42.0	647.6 ± 43.6	663.2 ± 50.5	563.7 ± 26.2^*^	0.132	0.175		
Change	75.2 ± 45.9		−99.5 ± 41.6				**0.008**	**0.027**
FFAs 30 min (μEq/L)^e^	304.6 ± 23.5	350.2 ± 30.3	334.8 ± 41.4	327.1 ± 32.6	0.986	0.652		
Change	45.6 ± 23.6		−7.77 ± 38.8				0.234	0.163
FFAs 60 min (μEq/L)^e^	251.2 ± 27.5	281.8 ± 30.6	251.6 ± 33.3	254.5 ± 40.3	0.935	0.268		
Change	30.6 ± 20.8		2.86 ± 27.7				0.421	0.400
FFAs 120 min (μEq/L)^e^	92.4 ± 4.40	84.5 ± 5.48	104.2 ± 13.9	88.1 ± 9.80	0.961	0.794		
Change	−7.92 ± 5.18		−16.1 ± 13.2				0.569	0.429
FFAs AUC (μEq/L×h)^e^	530.0 ± 29.2	590.6 ± 30.4	574.0 ± 39.2	539.3 ± 34.5	0.401	0.237		
Change	60.6 ± 33.5		−34.7 ± 30.8				**0.044**	0.066
Insulin 0 min (μIU/dL)^e^	9.92 ± 0.55	9.52 ± 0.42	10.1 ± 0.84	9.43 ± 0.80	0.969	0.558		
Change	−0.40 ± 0.50		−0.71 ± 0.51				0.662	0.592
Insulin 30 min (μIU/dL)^e^	44.0 ± 5.01	41.4 ± 3.88	51.6 ± 5.87	60.0 ± 9.25	0.330	0.116		
Change	−2.60 ± 3.73		8.42 ± 6.10				0.120	0.086
Insulin 60 min (μIU/dL)^e^	52.6 ± 5.38	49.8 ± 5.60	64.6 ± 6.16	59.4 ± 6.51	0.156	0.268		
Change	−2.76 ± 4.34		−5.24 ± 5.96				0.734	0.777
Insulin 120 min (μIU/dL)^e^	64.5 ± 8.07	58.3 ± 6.49	68.7 ± 11.6	64.7 ± 9.05	0.912	0.661		
Change	−6.16 ± 7.48		−4.02 ± 7.53				0.842	0.869
Insulin AUC (μIU/dL×h)^e^	96.2 ± 7.62	89.6 ± 6.46	111.1 ± 10.6	109.2 ± 10.7	0.349	0.143		
Change	−6.55 ± 5.30		−1.91 ± 7.14				0.599	0.290
Hemoglobin A1c (%)^e^	5.96 ± 0.07	5.99 ± 0.07	5.99 ± 0.14	5.98 ± 0.13	0.909	0.852		
Change	0.03 ± 0.03		−0.01 ± 0.05				0.454	0.459
C-peptide (μEq/L)^e^	2.16 ± 0.12	2.10 ± 0.09	2.21 ± 0.16	2.22 ± 0.16	0.977	0.764		
Change	−0.06 ± 0.08		0.01 ± 0.09				0.550	0.493

Mean ± SE; *AUC* area under the curve, *FFA* free fatty acid

^*^p < 0.05, ^**^p < 0.01, ^***^p < 0.001 derived from the paired *t*-test

^a^*p*-Values derived from the independent *t*-test for baseline values

^b^*p*-Values derived from the independent *t*-test for follow-up values

^c^*p*-Values derived from the independent *t*-test for change values

^d^*p*-Values adjusting for baseline

^e^Tested by logarithmic transformation

The 12-week intervention with Jerusalem artichoke and fermented soybean powder mixture supplementation resulted in a significant reduction in the HOMA-IR (*p* = 0.018) (Fig. 2). FFA serum levels at 0 min in the test group exhibited a significant decrease after the 12-week intervention (*p* = 0.034) (Table 2). The Δ serum level of FFAs at 0 min (*p* = 0.008) and the FFA AUC (*p* = 0.044) in the test group significantly differed from the placebo group, furthermore, these significances were maintained after adjusting for baseline values. However, no significant differences in insulin levels at any time point and insulin AUC were noted between the placebo and test groups (Table 2).

### The effects of 12 weeks of supplementation with a Jerusalem artichoke and fermented soybean powder mixture on oxidative stress markers

**Fig. 3:**
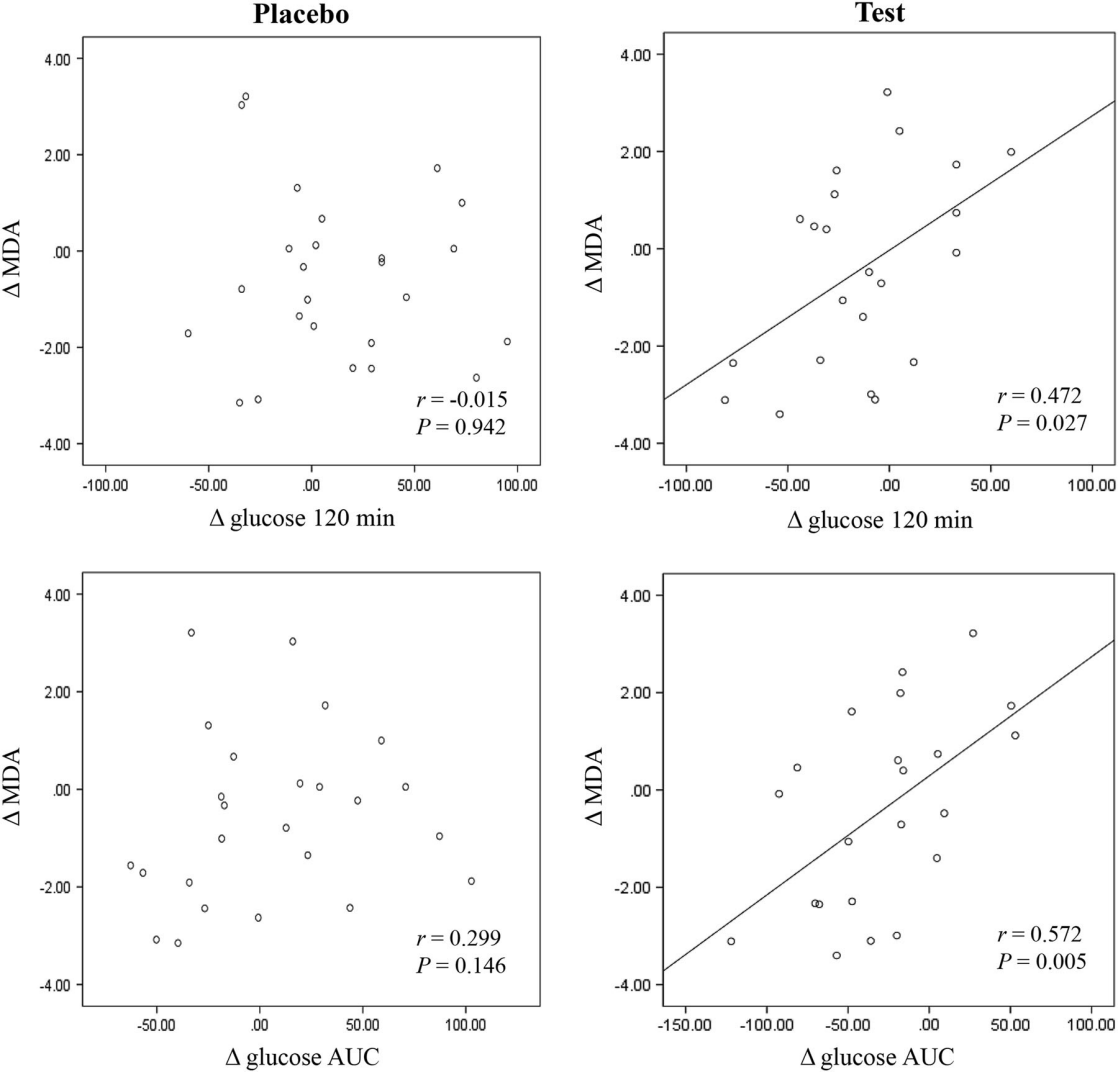
Correlation between changes (Δ, difference from baseline) in blood glucose levels and plasma MDA in the placebo and test groups. *r* Pearson’s correlation coefficients

After 12 weeks of treatment, the test group exhibited a significant decrease in urinary 8-epi-PGF_2α_ levels (*p* = 0.028), and the placebo group exhibited a significant increase in the urinary 8-epi-PGF_2α_ level (*p* = 0.040) (Fig. 2). The Δ urinary 8-epi-PGF_2α_ was significantly different between the two groups before (*p* = 0.003) and after adjusting for baseline value (*p* = 0.022). In the test group, the Δ glucose at 120 min was positively correlated with the Δ MDA (*r* = 0.472, *p* = 0.027), and the Δ glucose AUC was positively correlated with the Δ MDA (*r* = 0.572, *p* = 0.005) (Fig. 3). However, no significant associations were observed between Δ MDA and Δ glucose 120 min or between Δ MDA and Δ glucose AUC in the placebo group.

## Discussion

The antidiabetic effects of Jerusalem artichoke and fermented soybeans have raised interest in recent years; a previous study demonstrated that the combination of Jerusalem artichoke and fermented soybean had complementary antidiabetic effects through the potentiation of insulinotropic action and the reduction of IR in diabetic rats^21^. Therefore, we hypothesized that these findings might be extrapolated to humans. This randomized, double-blind, placebo-controlled study evaluated the effect of supplementation with a Jerusalem artichoke and fermented soybean powder mixture on glucose control in subjects with IFG, IGT, or newly diagnosed type 2 diabetes. As a result, the primary outcome (PPG level at 30 min; a time point when blood glucose levels increased promptly and rapidly in response to food intake) did not show any significant changes. However, changes in the PPG level at 60 and 120 min showed greater decrease in the test group than in the placebo group; finally, the glucose AUC was significantly reduced in the test group. Thus, overall glucose metabolism was improved, although the test product was not effective at slowing the prompt glucose absorption. Moreover, decreased blood glucose and HOMA-IR levels in the test group revealed the antidiabetic effects of Jerusalem artichoke and fermented soybeans in human subjects. In addition, diminished FFA levels and 8-epi-PGF_2α_ in the test group and the positive correlation between glucose and MDA levels suggested possible mechanisms related to the antidiabetic effects of Jerusalem artichoke and fermented soybeans.

The main component of Jerusalem artichoke is fructans, specifically inulin and fructooligosaccharides, which are dietary fiber that slow down absorption of blood glucose^22^ and are not absorbed in the intestines^23^. Therefore, consumption of Jerusalem artichoke did not affect insulin levels, and the present study revealed that supplementation with the Jerusalem artichoke and fermented soybean powder mixture did not affect insulin levels. Because of this absorption inhibition effect, we set a PPG level at 30 min, a time point that blood glucose increases rapidly, as a primary outcome rather than fasting glucose levels or PPG level at 120 min. Indeed, Chamukuttan et al.^24^ demonstrated that elevated PPG level at 30 min during OGTT was independently associated with incident diabetes in Asian Indians population; and Kabaroğlu et al.^25^ indicated that increased PPG level at 30 min (>140 mg/dL) during OGTT was related to higher systemic inflammatory levels in obese adolescents with normal glucose intolerance (fasting glucose < 100 mg/dL and PPG level at 120 min < 140 mg/dL). Thus, not only fasting glucose or PPG level at 120 min but also PPG level at 30 min becomes an important factor for regulating the risk of diabetes or diabetic-related disease. Moreover, in Korea, inulin is approved as an official functional material by Ministry of Food and Drug Safety; it stipulates that inulin has a function on improvement of PPG level. Based on the evidences, we assessed 30 min glucose in OGTT as a primary outcome. In the present study, although significant changes were not observed in 30 min glucose, changes in the PPG level at 60 and 120 min were significantly decreased in the test group than in the placebo group. Furthermore, the glucose AUC showed significant reduction in the test group. Thus, overall glucose metabolism was improved. Several studies also reported a beneficial effect of inulin on fasting glycemic control. A previous study demonstrated that fructooligosaccharide supplementation reduced fasting plasma glucose levels after 2 weeks in type 2 diabetes patients^26^. Another clinical trial demonstrated an antidiabetic effect of inulin-enriched pasta in humans, which reduced fasting glucose, HbA1c, and HOMA-IR levels^27^. In vivo studies have reported that Jerusalem artichoke supplementation improved IR and decreased the results of the OGTT. In addition, plasma glucose levels decreased with increasing levels of supplemented Jerusalem artichoke^28,29^. Inulin and fructooligosaccharides might affect glucose levels by changing the secretion of glucagon-like peptide-1, which increases insulin secretion, delays gastric emptying, promotes *β*-cell proliferation, and reduces *β*-cell apoptosis^30–34^. The mechanism of the hypoglycemic effect of Jerusalem artichoke remains largely unknown.

In clinical trials, the consumption of an inulin-rich product reduced HbA1c levels over 5 weeks^27^, and the consumption of a fermented soybean product decreased fasting blood glucose and HbA1c levels after 3 months in subjects with borderline and mild type 2 diabetes^35^. However, in the present study, HbA1c levels did not change during the study period. According to several studies, HbA1c levels show a poor correlation with the prediabetes status^36–38^. Since most of our study subjects displayed IFG levels or IGT and only a few individuals with newly diagnosed type 2 diabetes were included in the study, HbA1c levels might not be a proper tool to evaluate the improvement in glucose control in the present study.

Previous studies have demonstrated that long-term consumption of fermented soybean attenuated IR and improved glucose homeostasis in a type 2 diabetic animal model^18,39^. The biologically active components of fermented soybean include isoflavonoids and small peptides that are associated with IR and glycemic control^40,41^. These findings suggest potential mechanisms to explain the antidiabetic effect of fermented soybeans. According to a previous study, isoflavonoids might have an antidiabetic effect through estrogen-like action. Estrogen reduces IR through inhibition of intestinal glucose uptake and prevention of glucose-induced lipid peroxidation by inhibiting the sodium-dependent glucose transporter^42^. In addition, estrogen is associated with the stimulation of liver fatty acid metabolism and suppression of hepatic glucose production^43^. FFAs cause IR by inhibiting insulin-stimulated glucose uptake. High levels of plasma FFAs are associated with an increase in diacylglycerol and activation of protein kinase C, leading to increased tyrosine phosphorylation of insulin receptor substrate-1 and resulting in increased insulin-stimulated glucose transport activity and IR^44,45^. The present study demonstrated that supplementation with a Jerusalem artichoke and fermented soybean powder mixture decreased FFAs and IR, and both of these effects are explained by the presence of isoflavonoids in fermented soybeans.

Type 2 diabetes is associated with oxidative stress, and lipid peroxidation is the main marker of oxidative stress that plays a major role in the pathogenesis of type 2 diabetes^46–48^. In addition, 8-epi-PGF_2α_ is a secondary end product of peroxidation that can impair *β*-cell function and lead to apoptosis^49^. Improved metabolic control reduced 8-epi-PGF_2α_ by 32% in type 2 diabetes patients, and a clinical trial reported that 8-epi-PGF_2α_ was three-fold higher in type 2 diabetes patients than in healthy individuals^50,51^. Several investigators reported that 8-epi-PGF_2α_ is a predictor of glycemic control and oxidation status in patients with type 2 diabetes^52^ and a reliable marker of IGT^53^. In the present study, we observed that 8-epi-PGF_2α_ levels were significantly decreased in the test group. Jerusalem artichoke and fermented soybean powder mixture supplementation reduced oxidative stress in subjects with IFG, IGT, or newly diagnosed type 2 diabetes. Close interrelationships between changes of glucose and MDA were demonstrated in the present study despite the lack of differences in MDA levels between the placebo and test groups as well as no significant changes after the 12-week intervention. MDA is a primary biomarker of free radical-mediated lipid damage and oxidative stress and is elevated in type 2 diabetes patients^54–56^. This previous finding could explain the positive correlation between Δ glucose and Δ MDA in the test group of this study, indicating that lipid peroxidation was related to glycemic control and was affected by Jerusalem artichoke and fermented soybean powder mixture supplementation. The present results indicate that inulin in Jerusalem artichoke and isoflavonoids in fermented soybeans might complementarily affect glucose control, and Jerusalem artichoke and fermented soybean powder mixture supplementation could ameliorate existing oxidative stress in subjects with IFG, IGT, or newly diagnosed type 2 diabetes.

There are several limitations of our study design. First, we specifically focused on Korean subjects with IFG, IGT, or newly diagnosed type 2 diabetes. Therefore, our data cannot be generalized to other ethnic groups or to severe type 2 diabetes patients. Second, the sample size is relatively small; thus, the results should be interpreted with caution. Third, the levels of any inflammatory, antioxidant, or HDL markers that may affect or be associated with the results of this study were not measured. Finally, we did not assess the dose-dependent effects of the test product; thus, the effects of various doses of the test product should be verified through further studies. Despite these limitations, Jerusalem artichoke and fermented soybean powder mixture supplementation exhibited antidiabetic and antioxidant effects. In conclusion, the consumption of a Jerusalem artichoke and fermented soybean powder mixture for 12 weeks was effective for reducing PPG and oxidative stress level, particularly 8-epi-PGF_2α_, in subjects with IFG, IGT, or newly diagnosed type 2 diabetes.

## Electronic supplementary material

Table S1
