# Supplementary material for: The effects of Jerusalem artichoke and fermented soybean powder mixture supplementation on blood glucose and oxidative stress in subjects with prediabetes or newly diagnosed type 2 diabetes
Source: Nutr Diabetes. 2018 Jul 19;8:42. doi: 10.1038/s41387-018-0052-y (PMC6053428; doi:10.1038/s41387-018-0052-y)
Supplement: Supplementary file 1 — Table S1 [file 41387_2018_52_MOESM1_ESM.docx]

**Table S1. Dietary food intake and total energy expenditure of study participants**

|  | **Total (*n*=47)** | | | | | | | | ***P*^a^** | ***P*^b^** | ***P*^c^** |
| --- | --- | --- | --- | --- | --- | --- | --- | --- | --- | --- | --- |
|  | **Placebo group (*n*=25)** | | | | **Test group (*n*=22)** | | | |  |  |  |
|  | **Baseline** | | **Follow-up** | | **Baseline** | | **Follow-up** | |  |  |  |
| Basal metabolic rate (kcal/d) | 1455.9 | ±51.1 | 1455.5 | ±52.8 | 1336.7 | ±61.6 | 1332.1 | ±59.3 | 0.140 | 0.126 |  |
| Change |  | -0.36 | ±8.41 |  |  | -4.59 | ±6.66 |  |  |  | 0.700 |
| Total energy expenditure (kcal/d) | 2104.8 | ±55.3 | 2107.5 | ±55.4 | 1983.4 | ±59.5 | 1995.4 | ±59.5 | 0.142 | 0.174 |  |
| Change |  | 2.69 | ±8.95 |  |  | 12.0 | ±11.9 |  |  |  | 0.530 |
| Total calorie intake (kcal/d) | 2158.9 | ±52.4 | 2163.2 | ±53.5 | 2100.1 | ±69.6 | 2100.3 | ±63.4 | 0.497 | 0.449 |  |
| Change |  | 4.28 | ±9.07 |  |  | 0.15 | ±14.5 |  |  |  | 0.805 |
| Carbohydrate (%) | 61.8 | ±0.15 | 61.8 | ±0.16 | 61.8 | ±0.17 | 62.1 | ±0.16 | 0.926 | 0.236 |  |
| Change |  | 0.02 | ±0.20 |  |  | 0.27 | ±0.24 |  |  |  | 0.424 |
| Protein (%) | 15.9 | ±0.07 | 16.1 | ±0.07 | 15.9 | ±0.07 | 15.9 | ±0.08 | 0.883 | 0.271 |  |
| Change |  | 0.14 | ±0.09 |  |  | 0.01 | ±0.11 |  |  |  | 0.354 |
| Fat (%) | 22.9 | ±0.23 | 22.5 | ±0.24 | 22.4 | ±0.25 | 22.5 | ±0.23 | 0.150 | 0.884 |  |
| Change |  | -0.41 | ±0.33 |  |  | 0.04 | ±0.32 |  |  |  | 0.333 |
| Cholesterol (mg) | 185.6 | ±2.12 | 184.1 | ±1.29 | 181.6 | ±2.20 | 184.7 | ±2.76 | 0.193 | 0.832 |  |
| Change |  | -1.51 | ±2.19 |  |  | 3.15 | ±2.49 |  |  |  | 0.165 |

Mean ± SE.^∮^tested by logarithmic transformation, *P^a^*-values derived from independent *t*-test in baseline. *P^b^*-values derived from independent *t*-test in follow-up. *P^c^*-values derived from independent *t*-test in changed value. *^*^P* <0.05, *^**^P* <0.01, *^***^P* <0.001 derived from paired *t*-test.
